# Phytochemical Composition and Antioxidant Capacity of Three Malian Medicinal Plant Parts

**DOI:** 10.1093/ecam/nep109

**Published:** 2011-06-18

**Authors:** François Muanda, Donatien Koné, Amadou Dicko, Rachid Soulimani, Chafique Younos

**Affiliations:** ^1^Chemistry Laboratory and Methodologies for the Environment, Paul-Verlaine University, 57078 Metz, France; ^2^Ethnobotanic, Pharmacology, Anxiety, Stress Oxidative and Bioactivity Laboratory, University P. Verlaine-Metz, Metz, France

## Abstract

This study evaluates the levels of total polyphenolic compounds in three Malian medicinal plants and determines their antioxidant potential. Quantitative and qualitative analysis of polyphenolics contained in plants extracts were carried out by RP-C18 RP–HPLC using UV detector. The antioxidant activity was determined by three tests. They are phosphomolybdenum, DPPH (2,2-diphenyl-1 picrylhydrazyl) and ABTS [2,2′-azino-*bis*(3-ethylbenzothiazoline-6-sulfonic)] tests. The total phenolic and the total flavonoid contents varied from 200 to 7600 mg 100 g^−1^ dry weight (dw), expressed as gallic acid equivalents and from 680 to 12 300 mg 100 g^−1^ dw expressed as catechin equivalents, respectively. The total anthocyanin concentrations expressed as cyanin-3-glycoside equivalent varied from 1670 to 28 388 mg 100 g^−1^ dw. The antioxidant capacity was measured by determining concentration of a polyphenolic (in mg ml^−1^) required to quench the free radicals by 50% (IC_50_) and expressed as vitamin C equivalent antioxidant capacity. The IC_50_ values were ranked between 2.68 and 8.80 **μ**g ml^−1^ of a solution of 50% (v/v) methanol in water. The uses of plants are rationalized on the basis of their antioxidant capacity.

## 1. Introduction

Several epidemiological studies suggest that plants rich in antioxidants play a protective role in health and against diseases [1], and their consumption lowered risk of cancer, heart disease, hypertension and stroke [2–4]. The major groups of phytochemicals that may contribute to the total antioxidant capacity of plant include polyphenols and vitamins (C and E). Phenolic compounds can be nonnutrients [5]. Phenolic compounds of plants are hydroxylated derivatives of benzoic acid and cinnamic acids and have been reported to possess antioxidative and anticarcinogenic effects. Phenolic compounds including flavonoids are important in plant defense mechanisms against invading bacteria and other types of environmental stress [5, 6]. Flavonoids have long been recognized to possess anti-inflammatory, anti-allergic, antiviral and antiproliferative activities [5–9]. Several reports indicate that the antioxidant potential of medicinal plants may be related to the concentration of their phenolic compounds which include phenolic acids, flavonoids, anthocyanins and tannins [10, 11]. These compounds are of great value in preventing the onset and/or progression of many human diseases [12]. The health-promoting effect of antioxidants from plants is thought to arise from their protective effects by counteracting reactive oxygen species [11]. Antioxidants are compounds that help delay and inhibit lipid oxidation and when added to foods tend to minimize rancidity, retard the formation of toxic oxidation products, help maintain the nutritional quality and increase their shelf life [13].

We have recently reported the evaluation of the antioxidant potential of some medicinal and dietary plants [14, 15] and the positive correlation between peripheral blood granulocyte oxidative status and level of anxiety in mice [15–17].

The objectives of this investigation are (i) to evaluate the level of total phenolics, flavonoids and anthocyanins in three sub-Saharian medicinal plants (*Daniella oliveri, Ficus capensis* and *Vitex doniana*) used for treating hypertension and considered as diuretic, anti-inflammatory, antipyretic and antipurulent agents (Table 1) and (ii) to evaluate total antioxidant potential by using vitamin C equivalent antioxidant capacity (VCEAC) tests.

## 2. Methods

### 2.1. Apparatus

The RP–HPLC analyses were performed with a Waters 600E pump coupled to a Waters 486 UV visible tunable detector and equipped with a Alltech Intertsil ODS column (RP C18 column size 4.6 mm × 150 mm; particle size, 5 *μ*m). In addition, spectrophotometer analyses were carried out with UV-Vis spectrophotometer (Cary 50 scan).

### 2.2. Chemicals

Folin-Ciocalteu's phenol reagent, aluminum chloride, catechin, gallic acid, *p*-coumaric acid, coumarin, rutin, protocatechic acid, vitamin acid, caffeic acid, isovitexin, chlorogenic acid, delphinidin, orientin, malvidin, homoorientin, ellagic acid, l-cyanidin, peonidin were purchased from Across Organics. Sodium carbonate, sodium nitrite, chlorhydric acid, ethyl acetate, sodium sulfate anhydrous, ammonium phosphate, ferric ammonium sulfate, acetonitrile, methanol, 2,2′-azino-*bis*(3-ethylbenzothiazoline-6-sulfonic) (ABTS), PBS buffer, AAPH [2,2′-azo-*bis*(2-amidino-propane)dihydrochloride; ABTS: 2,2′-azino-*bis*(3-ethylbenzothiazoline-6-sulfonic)] and DPPH (2,2-diphenyl-1 picrylhydrazyl) were obtained from Sigma and Roth (France). The chemicals used were all of analytical grade.

### 2.3. Procurement and Preparation of Samples

The plants *D. oliveri*, *F. capensis* and *V. doniana* were obtained from the Department of Traditional Medicine of Mali, upon arrival at the laboratory, different parts of the plants (leaves, root barks and stem barks) were dried at room temperature, powdered and sifted in a sieve (0.750 *μ*m). The plant material was biologically authenticated by the National Institute for Research in Public Health of Bamako.

### 2.4. Samples Extractions

#### 2.4.1. Total Phenolic, Flavonoid, Anthocyanin Contents and Antioxidant Capacity

Samples for total phenolic compounds (TPC), total flavonoid compounds (TFC), total anthocyanin compounds (TAC) and total antioxidant capacity assays were extracted from the different powders as described by Makkard et al. [27] slightly modified. The powder sample (2 g) was extracted twice with 20 ml of cold aqueous methanol solution (50%). The two volumes were combined, made up to 40 ml, centrifuged at 1238 g for 20 min and transferred in small sample bottles and stored at +4°C in the dark until analysis.

#### 2.4.2. Extraction of Polyphenol Compounds for RP–HPLC Analysis

Polyphenols were extracted following the method described by Muchuweti et al. [28] slightly modified. Fresh samples (5 g) of plants portions were extracted twice with ethyl acetate (20 ml) and organic fractions were combined. After 30 min of drying with anhydrous NaSO_4_, the extract was evaporated to dryness at 40°C. Then, the residue was dissolved in methanol/water [2 ml 1 : 1(v/v)] before analysis by RP–HPLC. The standard solutions were prepared by dissolving 1 mg ml^−1^ (m/v).

### 2.5. Dosage of Phenolic Compounds

#### 2.5.1. Spectrophotometer Analysis

**Dosage of TPC**

TPC were determined following Muchuweti et al. [28] method which was slightly modified. To a sample of 100 *μ*l, distilled water was added to make the quantity 2 ml (Eppendorff tube), followed by addition of 1 ml of Folin-Ciocalteu reagent (1N) and sodium carbonate (20%). After 40 min at room temperature, absorbance at 725 nm was read on a spectrophotometer against a blank that contained methanol instead of sample. TPC were expressed in terms of equivalent amounts of gallic acid (GAE).

**Determination of TFC**

TFCs were measured according to a colorimetric assay slightly modified [12, 29]. A 250 *μ*l of standard solution of catechin at different concentrations or appropriately diluted samples was added to 10 ml volumetric flask containing 1 ml of didistillate waters (ddH_2_O). At time 0 min, 75 *μ*l of NaNO_2_ (5%) was added to the flask. After 5 min, 75 *μ*l of AlCl_3_ (10%) was added. At 6 min, 500 *μ*l of NaOH (1N) was added to the mixture. Immediately, the solution was diluted by adding 2.5 ml ddH_2_O and mixed thoroughly. Absorbance of the mixture, pink in color, was determined at 510 nm versus the prepared blank. TFCs in medicinal plants were expressed as microgram-catechin equivalents (CE)/gram dry weight (dw). Samples were analyzed in three replications.

**Evaluation of TAC**

The anthocyanin contents of samples was estimated by a UV-spectrophotometer with the pH-differential method [30, 31] using two buffer systems, potassium chloride buffer, pH 1.0 (0.025 M) and sodium acetate buffer, pH 4.5 (0.4 M). Briefly, 400 *μ*l of extract was mixed in 3.6 ml of corresponding buffer solutions and read against a blank at 510 and 700 nm. Absorbance (Δ*A*) was calculated as: Δ*A* = (*A*_510_ − *A*_700_) pH_1.0_ − (*A*_510_ − *A*_700_) pH_4.0_ [30–32]. Monomeric anthocyanin pigment concentration in the extract was calculated and expressed as cyaniding −3 glycoside (mg l^−1^): Δ*A* × MW × Df × 1000/(Ma × 1)  [30–33] with Δ*A*: Absorbance, Mw: molecular weight (449.2), Ma: Molecular absorptivity (26.900) and Df: dilution factor.

#### 2.5.2. RP–HPLC Analysis

RP-RP–HPLC analysis was performed according to the modified method describe [34, 35]. Extracted sample was filtered through a 0.45-*μ*m polytetrefluoroethylene syringe tip filter, using a 20-*μ*l sample loop. The sample was analyzed using an RP–HPLC system equipped with a waters UV-Visible tunable detector on a Reverse Phase (RP C18) column Alltech Intertsil ODS-5 *μ*m × 4.6 mm × 150 mm. The flow rate was set at 1 ml min^−1^ at room temperature. A gradient of three mobile phases was used in the study, solvent A: 50 mM ammonium phosphate (NH_4_H_2_PO_4_) pH 2.6 (adjusted with phosphoric acid); solvent B: Which was constituted of 80 : 20 (v/v) acetonitrile/solvent A, and solvent C, constituted of 200 mM phosphoric acid pH 1.5 (pH adjusted with ammonium hydroxide). The solvents were filtered through a Whatman Maidstone England paper No. 3 and putted in an ultrasonic apparatus for 25 min. The gradient profile was linearly change as follows (total 60 min): 100% solvent A at 0 min, 92% A/8% B at 4 min, 14% B/86% C at 10 min, 16% B/84% C at 22.5 min, 25% B/75% C at 27.5 min, 80% B/20% C at 50 min, 100% A at 55 min, 100% A at 60 min [36]. After each run, the system was reconditioned for 10 min before analysis of next sample. Under these conditions, 20 *μ*l of sample were injected. All sample analysis was done in triplicate. Polyphenolic standards prepared by dissolving 1 mg ml^−1^ were used to generate characteristic UV spectra and calibration curves. The individual polyphenolic compounds in the sample were identified by comparison of their UV-visible spectra and their retention times with the spike of the corresponding polyphenolic standards.

The detection was carried out at 280 and 320 nm and their quantification was obtained by the comparison of the peaks area with the corresponding standards calibration curves. Collected results were reported as equivalent amount of commercial standard.

### 2.6. Antioxidant Activity

Three different tests have been used to determine the total antioxidant capacity: the phosphomolybdenum (PPM) test, the ABTS test and the DPPH test [37, 38].

#### 2.6.1. PPM Test

The PPM assay is a DPPH scavenging method in which, hydrogen and electron transfer from antioxidant analytes to DPPH and Molybdenum(VI) complex occur in the DPPH and PPM. The transfers occur at different redox potentials in the two assays and also depend on the structure of antioxidant. Several flavonoids and phenols have been isolated from plant parts with potent DPPH scavenging activities [39], whereas the PPM method usually detects antioxidants such as vitamins C, E and some specific phenol [37]. In general, the extraction solvent affects the antioxidant capacity, the aqueous methanol extract showed better antioxidant activities than the organic extract, aqueous alcohol is considered to be the best solvent for the extraction of phenolic compounds from plant materials [40, 41].

The total antioxidant capacity of the plant extracts was measured by the method described by Prieto et al. [37]; 100 *μ*l of the sample solution was mixed with 900 *μ*l of the reagent solution (0.6 M sulfuric acid, 28 mM sodium phosphate and 4 mM ammonium molybdate) against a blank containing 100 *μ*l of methanol mixed with 900 *μ*l of reagent solution. The absorbance of the test sample was measured at 695 nm. The antioxidant activity was expressed as vitamin C equivalent (mg 100 g^−1^ dry matter).

#### 2.6.2. ABTS Test

The method used in this test is the one developed by Vanden Berg et al. [38], slightly modified. One millimolar of AAPH solution was mixed with 2.5 mM ABTS as diammonium salt in phosphate buffered saline (PBS) solution 100 M potassium phosphate buffered (pH 7.4) containing 150 mM NaCl. The mixture was heated in a water bath at 68°C for 20 min. The concentration of the resulting blue-green ABTS radical anion solution was adjusted to an absorbance of 0.65 ± 0.02 at 734 nm. The sample solution (60 *μ*l) was added to 2.94  ml of the resulting blue–green ABTS radical solution. The mixture, protected from light, was incubated in a water bath at 37°C for 20 min. Then the decrease of absorbance was measured at 734 nm. The control solution was consisted by 60 *μ*l of methanol and 2.94 ml of ABTS radical anion solution. The stable ABTS radical anion scavenging activity of the plants phenolic compounds in the extracts was expressed as mg 100 g^−1^ dry plants powders and as mg 100 ml^−1^ standards compounds of VCEAC in 20 min. All radical stock solutions were prepared fresh daily.

#### 2.6.3. DPPH Test

**DPPH Evaluation**

The antioxidant activity of plant extract was estimated using a slight modification of the DPPH radical scavenging protocol reported by Chen et al. [42]; 1 ml of 100 *μ*M DPPH solution in methanol was mixed with 0.1 ml of plant extract. The reaction mixture was incubated in the dark for 20 min and thereafter the optical density was recorded at 517 nm against the blank.

For the control, 1 ml of DPPH solution in methanol (100 *μ*M) was mixed with 0.1 ml of methanol and optical density of the solution was recorded after 20 min. The decrease in optical density of DPPH on addition of test samples in relation to the control was used to calculate the antioxidant activity as percentage of inhibition (%IP) of DPPH radical, %IP = [(*At*_0_ − *At*_20_)/(*At*_0_ × 1000)] [12, 43] where *At*_0_: absorbance of the sample test after 0 min and *At*_20_: absorbance of the control after 20 min. Each assay was carried out in triplicate.

From a plot of concentration against %IP, a linear regression analysis was performed to determine the IC_50_ value (concentration of a polyphenolic (in mg ml^−1^) required to quench the free radicals by 50%) for each plant extract. The DPPH radical scavenging activity of phenolic compounds was expressed as IC_50_ value in micrograms per milliliter of fresh weight. A low IC_50_ value represents a high antioxidant activity.

**DPPH Determination**

The DPPH scavenging activity was determined using a modified method of Kim et al. [35]. To 2.90 ml of an aqueous methanol solution (50%) of 100 *μ*M of DPPH, 100 *μ*l of the plant extracts solution was added. The mixture was shaken and allowed to stand at 20°C in dark for 30 min. After the decrease in absorbance, the resulting solution was monitored at 517 nm. The DPPH radical scavenging activity of phenolic compounds was expressed as mg 100 g^−1^ of dry matter and as mg 100 ml^−1^ of VCEAC in 30 min. The control solution was consisted by 100 *μ*l of methanol and 2.90 ml of DPPH solution. The radical solution was prepared daily.

### 2.7. Statistical Analysis

Results are presented as mean ± standard error; statistical analysis of experimental result was based on analysis of variance. Significant difference was statistically considered at the level of *P* < .001.

## 3. Results

### 3.1. TPCs, TFCs and TACs

**Figure 1:**
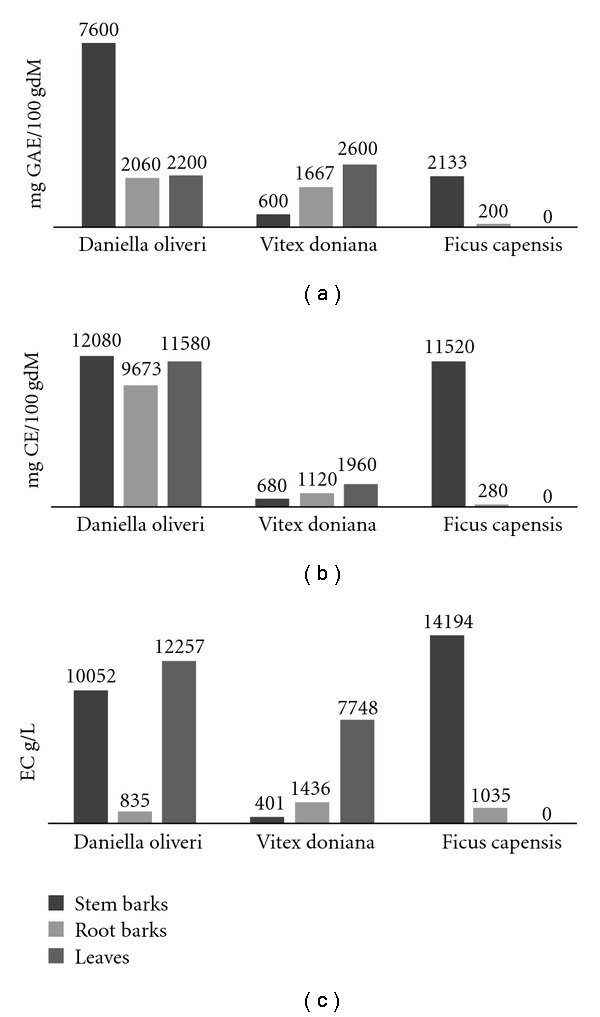
(a) Total polyphenols, (b) total flavonoids, (c) total anthocyanins.

TPCs, TFCs and TACs were quantified using a UV-vis spectrophometric apparatus. The results of analysis are showed in Figure 1. No data were recorded for *F. capensis* leaves due to lack of sample.

### 3.2. RP–HPLC Analysis

Quantitative and qualitative comparison of polyphenolic compounds (TPC, TFC, TAC) were conducted using RP–HPLC.

The retention time of standards and their corresponding concentration in the samples were collected in Table 2. The experimentation has been done in four replicates. However, it is important to note that numerous peaks were not identified owing to the absence of suitable standards.

### 3.3. Antioxidant Activity

**Figure 2:**
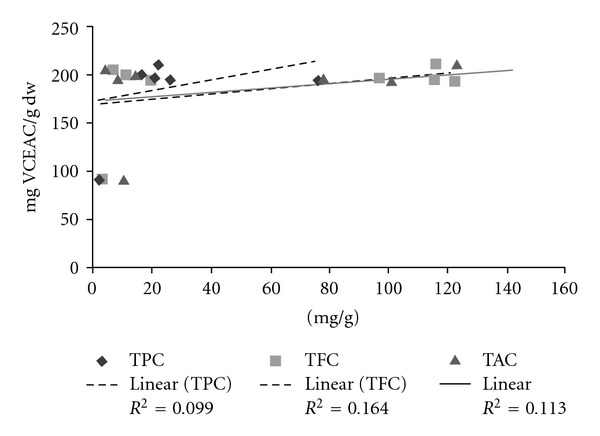
Relationship between the antioxidant activities and the polyphenolic compounds TPC (Total Phenolic Compounds); TFC (Total Flavonoid compounds) and TAC (Total Anthocyanin Compounds).

On the three plants screened, the extracts revealed good scavenging antioxidant activities as well as by PPM, ABTS or DPPH tests. The scavenging antioxidant activities of the different samples were reported in Table 3.  Figure 2 showed the relationship between the antioxidant activities and the polyphenolic compounds (TPC, TFC, TAC) in the samples.

## 4. Discussion

The distribution of TPC in *D. oliveri* and *V. doniana* differs. The content of TPC are higher in leaves than in stem barks in *V. doniana*, whereas in *D. oliveri* TPC is more concentrated in the stem barks (Figure 1). The concentration of TFC is very low in the root barks of *F. capensis*. The stem bark extracts of *D. oliveri* and *F. capensis* contain almost the same levels of TFC. *Daniella oliveri* plant parts, stem barks, root barks and leaves exhibit a similar TFC (Figure 1). For all the three plants, the concentration of TAC is lowest in the root barks.

RP–HPLC analysis revealed that the caffeic acid in the stem barks of *D. oliveri* is the most important phenolic compound (2410.4 *μ*g ml^−1^), whereas its levels are too low in the other two plants (*V. doniana*, 8.2 *μ*g ml^−1^ and *F. capensis*, 12.7*μ*g ml^−1^). Moreover, it appears that rutin is in very high concentration (6363.0 *μ*g ml^−1^) in the root barks of *V. doniana* and almost absent in the root barks of *D. oliveri* and *F. capensis*.

Rutin is the most important phenolic compound (11943.0 *μ*g ml^−1^) in the leaves of *V. doniana*, while it is not detected in the leaves of *D. oliveri* (Table 2).

Antioxidant activity has been evaluated by three tests: PPM, ABTS and DPPH. The PPM assay showed that the highest value was 606.0 mg 100 g^−1^ dw (VCEAC) for the root barks of *D. oliveri*; in contrast, the lowest one was 60.0 mg 100 g^−1^ dw for the root barks of *F. capensis* (Table 3). The great variations observed between the different plants and plant parts could be explained by the fact that PPM essay evaluates the antioxidant activity of polyphenols, and others antioxidant agents which are not phenolic compounds [43]. To be more accurate about phenolic compounds, ABTS and DPPH tests have been done. ABTS tests showed that the antioxidant activity of different plants was almost the same. DPPH tests expressed as VCEAC varied from 91.3 mg 100 g^−1^ dw for the root barks of *F. capensis* to 205.5 mg 100 g^−1^ dw for the stem barks of *V. doniana*. In addition, the antioxidant activity evaluated as %IP revealed a similar behavior. The highest IP value was 93.3% for the stem barks of *V. doniana* and the lowest one was 28.4% for the root barks of *F. capensis*. The %IP and IC50 (*μ*g ml^−1^) have been calculated to compare the antioxidant capacity of the studied plant parts extracts with those described by other authors in literature such as Adesegun et al. [44] and Ruchi et al. [43]. %IP values were relatively high (28.41–93.3%) and IC_50_ relatively weak (2.7–8.8 *μ*g ml^−1^). This revealed that these three Malian plants have very good antioxidant activities. Each plant contains generally different phenolic compounds with different amount of antioxidant activity.

Many studies indicate linear relationship between total phenolics and antioxidant activity [10, 12, 45]. In this study we found that polyphenolic compounds were not major contributors to antioxidant activity, since for TPCs, TFCs and TACs versus antioxidant activity, the correlation coefficients *R* ^2^ = 0.0998, 0.1641, 0.1135, respectively, were weak (Figure 2). These correlations have been established using all plant parts (stem barks, root barks, leaves). In conclusion, our results suggest that these plants are strong radical scavengers and can be seen as potential source of natural antioxidants for medicinal and commercial uses.

## Funding

Ministry of Scientific Research of the Republic Democratic of Congo grant (No. 132.49/060/KMB/07).

## Figures and Tables

**Table 1:** Name, traditional uses and phytocomponents data.

Plant name	Family	Uses	Pharmacology data	Phytocomponents data
*Daniella oliveri* (*D. thurifera*) Rolfe	Caesalpiniaceae	Treatment diarrheic (leaves), Bactericide, anti-inflammatory, analgesic, antiseptic, anti-diabetic, antispasmodic, anti-haemorrhoid, aphrodisiac, relaxing	Analgesic (hexane extract), antipyretic (ethyl acetate extract), anti-inflammatory, bactericide, anti-histamic (methanol extract) [18–21]	Polyphenols, flavonoids, anthocyanins, glycosides, tannins, saponins, terpenes, alkaloids

*Vitex doniana* (*V. umbrosa*)	Verbenaceae	Bactericide (leaves and stems); diuretic (leaves) tonifiant (roots); aphrodisiac (leaves, roots) [22, 23]; anti-diabetic (stems) antiseptic (leaves)	Bactericide (aqueous extract)	Saponins, steroids, terpene, [24] flavonoids, polyphenols, vitamins C, A, E

*Ficus capensis* (Thumb) (Forssk)	Moraceace	Bactericide, anti-diabetic, diuretic, aphrodisiac (stems, roots) [20, 25, 26]	Anti-diabetic, diuretic (methanol extract)	Polyphenols, flavonoids, tannins, vitamin C

**Table 2:** Compounds identified in the different plant parts and their concentration.

Name of compound	Family	Retention time (min)	Stem barks (*μ*g ml^−1^)			Root barks (*μ*g ml^−1^)			Leaves (*μ*g/ml)	
			*D. oliveri*	*V. doniana*	*F. capensis*	*D. oliveri*	*V. doniana*	*F. capensis*	*D. oliveri*	*V. doniana*
Gallic acid	P	11.2	210.1 ± 1.5	190.9 ± 0.2	1180 ± 4	1202 ± 2	168.6 ± 0.4	1.6 ± 0.1	292.5 ± 0.3	471.4 ± 0.2
Protocatechic acid	P	17.0	19.8 ± 0.2	63.5 ± 1.4	71.6 ± 0.3	1.2 ± 0.1	22.7 ± 0.1	1.6 ± 0.1	0.8 ± 0.1	34.8 ± 0.3
Catechin	F	25.0	ND	10.4 ± 0.1	3.0 ± 0.1	ND	51.5 ± 0.2	0.8 ± 0.1	4.1 ± 0.1	1.4 ± 0.1
Chlorogenic acid	P	26.5	505.2 ± 0.4	4.2 ± 0.1	12.3 ± 0.1	ND	ND	0.6 ± 0.1	1.1 ± 0.1	1.7 ± 0.1
Caffeic acid	P	28.7	2410.4 ± 12	8.2 ± 0.1	12.7 ± 0.1	0.9 ± 0.1	ND	5.2 ± 0.1	13.6 ± 0.2	ND
*p*-Coumaric acid	P	33.5	322.4 ± 3.7	9.2 ± 0.1	827.2 ± 3.5	127.6 ± 2.1	ND	827.2 ± 0.8	18.9 ± 0.2	18.8 ± 0.3
Homo-orientin	F	35.4	784.4 ± 4.9	453.6 ± 4.0	36.6 ± 0.1	6.2 ± 0.2	2804 ± 4	194.9 ± 0.3	894.9 ± 4.5	384.1 ± 2
Orientin	F	36.4	ND	3.8 ± 0.1	9.0 ± 0.1	1.0 ± 0.1	247.1 ± 2.0	9.0 ± 0.1	ND	1.0 ± 0.2
Rutin	F	37.1	144.2 ± 2.4	34.9 ± 0.2	22.7 ± 0.2	1.0 ± 0.1	6363 ± 2	6.1 ± 0.1	ND	11943 ± 5
Quercitrin-glucosyl	F	38.0	224.1 ± 0.7	96.3 ± 0.3	ND	115.6 ± 0.4	18.1 ± 0.1	ND	12.3 ± 0.2	12.6 ± 1
Quercitrin dehydrate	F	39.3	5.0 ± 0.2	78.7 ± 0.2	1.8 ± 0.1	22.4 ± 0.1	1346 ± 1	83.2 ± 0.5	ND	1.7 ± 0.1
Coumarin	P	40.4	1.9 ± 0.1	2.5 ± 0.1	13.8 ± 0.1	4.9 ± 0.1	33.9 ± 0.7	2.9 ± 0.1	29.2 ± 0.4	10.9 ± 0.1
Malvidin	A	42.0	ND	39.1 ± 0.2	ND	ND	110.0 ± 0.6	ND	ND	8.3 ± 0.1
Delphinidin	A	42.5	1.1 ± 0.1	35.3 ± 0.1	34.4 ± 0.1	ND	ND	7.6 ± 0.1	ND	ND
Quercitrin	F	44.0	1.0 ± 0.1	ND	109.5 ± 1.0	5.0 ± 0.1	323.2 ± 0.1	63.3 ± 0.2	ND	1831 ± 18
Ascorbic acid	Vit. C	56.5	2.5 ± 0.1	1.6 ± 0.1	14.5 ± 0.4	4.0 ± 0.1	ND	1.3 ± 0.1	ND	ND

ND: not determinate; A: Anthocyanidins; F: Flavonoids; P: Polyphenol. Data were reported as mean ± SEM (*n* = 4).

**Table 3:** Antioxidant activity *in vitro* analysis.

Plants	Parts	Test PPM (mg 100 g^−1^ dw)	Test ABTS (mg 100 g^−1^ dw)	Test DPPH		
				VCEAC (mg 100 g^−1^ dw)	% IP	IC_50_ (*μ*g ml^−1^)
*Daniella oliveri*	Stem barks	586 ± 12	127.5 ± 0.1	193.7 ± 1.8	86.1 ± 1.4	2.9 ± 0.1
	Root barks	606 ± 1	124.1 ± 0.9	196.3 ± 0.7	87.6 ± 0.3	2.8 ± 0.1
	Leaves	526 ± 4	109.2 ± 3.8	210.3 ± 0.4	93.3 ± 0.2	2.7 ± 0.1
*Vitex doniana*	Stem barks	74 ± 6	129.6 ± 0.1	205.5 ± 2.3	84.9 ± 1.3	2.9 ± 0.1
	Root barks	194 ± 7	126.2 ± 0.9	200.1 ± 1.1	87.7 ± 0.1	2.8 ± 0.1
	Leaves	180 ± 5	127.1 ± 0.1	195.0 ± 1.3	84.9 ± 0.7	2.9 ± 0.1
*Ficus capensis*	Stem barks	280 ± 3	120.8 ± 6.1	195.8 ± 3.3	85.40 ± 1.80	2.9 ± 0.1
	Root barks	60 ± 2	122.5 ± 1.4	91.3 ± 0.5	28.41 ± 0.23	8.8 ± 0.1
